# Nicotine and cotinine quantification after a 4-week inhalation of electronic cigarette vapors in male and female mice using UPLC-MS/MS

**DOI:** 10.15537/smj.2022.43.7.20220142

**Published:** 2022-07

**Authors:** Fawaz Alasmari, Abdullah F. Alasmari, Ehab Elzayat, Majed M. Alotaibi, Farraj M. Alotaibi, Mohamed W. Attwa, Fars K. Alanazi, Elkhatim H. Abdelgadir, Syed Rizwan Ahmad, Faleh Alqahtani, Salim S. AL-Rejaie, Musaad A. Alshammari

**Affiliations:** *From the Department of Pharmacology and Toxicology (F. Alasmari, F. M. Alotaibi, A. F. Alasmari, Alqahtani, AL-Rejaie, Alshammari); from the Department of Pharmaceutics (Elzayat, Alanazi); from the Department of Pharmaceutical Chemistry (Attwa, Ahmad), College of Pharmacy, King Saud University, and from the Department of Forensic Sciences (M. M. Alotaibi, Abdelgadir), College of Criminal Justice, Naif Arab University for Security Sciences, Riyadh, Kingdom of Saudi Arabia.*

**Keywords:** electronic cigarettes, nicotine and cotinine, gender differences, biological detection, UPLC-MS/MS

## Abstract

**Objectives:**

To detect the cotinine and nicotine serum concentrations of female and male C57BL/6J mice after a 4-week exposure to electronic (e)-cigarette vapors using ultra-performance liquid chromatography-tandem mass spectrometry (UPLC-MS/MS).

**Methods:**

This experimental study was carried out at an animal facility and laboratories, College of Pharmacy, King Saud University, Riyadh, Saudi Arabia, between January and August 2020. A 4-week exposure to e-cigarettes was carried out using male and female mice and serum samples were obtained for cotinine and nicotine quantification using UPLC-MS/MS. The chromatographic procedures involved the use of a BEH HSS T3 C18 column (100 mm x 2.1 mm, 1.7 μm) with acetonitrile as a mobile phase and 0.1% formic acid (2:98 v/v).

**Results:**

The applied methodology has highly efficient properties of detection, estimation, and extraction, where the limit of quantification (LOQ) for nicotine was 0.57 ng/mL and limit of detection (LOD) for nicotine was 0.19 ng/mL, while the LOQ for cotinine was 1.11 ng/mL and LOD for cotinine was 0.38 ng/mL. The correlation coefficient was r^2^>0.99 for both compounds. The average recovery rate was 101.6±1.33 for nicotine and 100.4±0.54 for cotinine, while the precision and accuracy for cotinine and nicotine were less than 6.1. The serum cotinine level was higher in males (433.7±19.55) than females (362.3±16.27).

**Conclusion:**

This study showed that the gender factor might play a crucial role in nicotine metabolism.

**T**he inhalation of tobacco products leads to high levels of nicotine in the human systems. These tobacco products include electronic and combustible cigarettes, cigars, hookah, and others. Importantly, electronic (e)-cigarette devices are battery-based devices and are operated to push nicotine and other chemicals such as flavoring agents into the environment.^1-3^ Electronic-cigarette devices are produced in different types such as tank devices, electronic cigars, electronic pipe, customizable e-cigarette mode, and others. E-liquids are activated by stimulating the internal heat source, which releases the vapors containing nicotine and other favoring reagents.

Electronic delivery systems of cigarettes in animal models have been recently developed. Several published works used computerized delivery machines to deliver e-cigarette vapors in the environments, and the pre-clinical models can then be exposed through inhalation.^1-4^ It has been found that these electronic systems are proper delivery techniques and can produce toxicological effects in pre-clinical models after exposure to concentrations of nicotine-containing vapors.^1-5^

The use of e-cigarettes is currently widespread in developed countries. The e-cigarette devices release vapors containing nicotine without the chemicals present in conventional cigarettes. The e-liquids usually contain water, nicotine, flavoring ingredients, vegetable glycerin (VG), and propylene glycol (PG), which are prepared in laboratories and factories. Evidence indicates that the urinary cotinine level is an essential biomarker for investigating any previous tobacco exposure.

A bioanalytical technology was validated and developed by Awwad et al^6^ to determine the levels of cotinine and nicotine in humans’ blood. This methodology used dried blood spots and liquid chromatography (LC)-Orbitrap mass spectrometry (MS) technology. A heated-electrospray ionization (ESI) source was used at positive ions, and it detected nicotine at m/z of 163.1235, and at m/z of 177.1028, and the internal standard at m/z of 166.1423. This study established a linear calibration curve for cotinine in the range of 10-500 ng/mL, while the range was 5-250 ng/mL for nicotine. Awwad et al^6^ reported that the accuracy of both cotinine and nicotine was above 85% for quality control and 80% for a lower limit of quantification, while the precision for both compounds ere within 15%. A study by Stolker et al^7^ identified an analytical methodology to detect both nicotine and cotinine in rats’ plasma using LC-QqQ-MS system (triple quadrupole system), and they found that the selection of ionization pairs (m/z) was reported as: cotinine= 177→98 (parent ion →MS-MS confirmation ion), and nicotine= 163→106 (parent ion →MS-MS confirmation ion).

Earla et al^8^ developed a methodology of electrospray ionization LC-MS. The method was fast, simple, and sensitive to the detection of nicotine, cotinine, trans-3ʹ-hydroxycotininenornicotine, and norcotinine using a strong cation solid-phase extraction. The samples were plasma obtained from human smokers, either human immunodeficiency virus (HIV)-1-positive or HIV-negative smokers. Importantly, the multiple reaction monitoring transitions calculated in m/z for nicotine: 163.3/117.1, cotinine: 177.5/80.3, nornicotine: 149.5/132.3, norcotinine: 163.4/80.3, and trans-3ʹ-hydroxycotinine: 193.2/80.1. This report showed that 0.53 ng/ml was the limit of quantification (LOQ) for nicotine and its metabolites, which is considered a highly sensitive value. Earla et al^8^ detected nicotine in the plasma ~5-fold higher in HIV-negative smokers (33.29±15.4 ng/ml) compared with HIV-positive smokers (7.17±3.8 ng/ml). However, the levels of nornicotine were ~3-fold lower for HIV-negative smokers (2.3±1.2 ng/ml) compared with HIV-positive smokers (6.8±2.9 ng/ml). Human immunodeficiency virus-positive smokers had non-significant higher plasma cotinine concentrations (85.6±60.5 ng/ml) as compared with HIV-negative smokers (74.9±40.5 ng/ml).

Beyer et al^9^ validated and developed 2 assays, liquid chromatographic/atmospheric pressure chemical ionization tandem mass spectrometric (LC-APCI-MS) and liquid chromatography-electrospray ionization tandem mass spectrometric (LC/ESI-MS/MS), for the quantification of cotinine, nicotine, and other compounds. Their study found that both LC-APCI-MS and LC-ESI-MS/MS showed improved selectivity properties for nicotine and cotinine. The instability was not observed with multiple freezing and thawing on the samples. The 2 assays showed linearity with a range of detection of 1-1000 ng/ml using LC-ESI-MS/MS, while the detection range was 50-1000 ng/ml using LC-APCI-MS technology. Liquid chromatographic/atmospheric pressure chemical ionization tandem mass spectrometric showed an accuracy of 38.6-14%, while LC-ESI-MS/MS showed an accuracy of 38.3-8.3%. The intermediate precision was 4.8-3.5% using the LC-APCI-MS system and 4.3-14.7% using LC-ESI-MS/MS methodology. Although LC-ESI-MS/MS showed more identification power and sensitivity advantages, both assays showed acceptable sensitivity and precise quantification properties (exception of nicotine of rather volatile). The levels of cotinine and nicotine were measured following published work.^7,10^ This present study used the ultra performance (UP)LC-MS/MS technique to investigate whether gender plays a crucial role in accumulating nicotine or cotinine following inhalation of e-cigarette vapors.

## Methods

This experimental study was carried out at an animal facility and laboratories, College of Pharmacy, King Saud University (KSU), Riyadh, Saudi Arabia, between January and August 2020. C57BL/6J wild type mice were obtained from the Animal Care Center, College of Pharmacy, KSU. The mice were maintained with 40-60% humidity, at a room temperature of 22±2^°^C, fed with standard rodent chow, and provided with water ad libitum. All experimental procedures were approved by the Institutional Animal Care and Use Committee, KSU (SE-19-130). The reason for using C57BL/6J mice is that a prior work demonstrated that a similar exposure to e-cigarettes induced alterations on the concentrations of the glutamate, glutamine, gamma-aminobutyric acid, and dopamine in the mesocorticolimbic system of C57BL/6J mice.^3^ A total of 3 male and 3 female mice, each weighing between 23-27 grams, were briefly acclimatized for 4 weeks and exposed to specific characteristics of e-cigarettes for 4 weeks. The mice inhaled vapors containing nicotine (25 mg/mL), 70/30 VG/PG, and berry ratio for 30 days. This e-cigarette mixture is used in e-cigarette brands. The mice were observed twice every day to check for any mortality and morbidity throughout the exposure. All mice were checked daily for the time and duration of clinical signs and symptoms. The mice were placed in whole-body chambers (cages) in a hood supplied with a ventilation system for one hour per day, 5 days per week, for 4 weeks. Following treatment, mice were anesthetized using both 50 mg/ml ketamine and 20 mg/ml xylazine. Then, their blood was collected, the mice were sacrificed, and the serum samples were obtained using a centrifuge apparatus.

A solution containing a 70/30 ratio of VG/PG was used in this study through an electric smoking inhalation system. Then, no nicotine for the vehicle control group, and 25 mg/mL of nicotine was added to the mixture for the e-cigarette-containing nicotine group.

A positive control e-liquid-containing identical ingredients to the one that we prepared in our laboratory was bought from the market. A total of 3 liquid samples (positive control e-liquid-containing 25 mg/mL nicotine [commercial product], e-liquid prepared in our laboratory without nicotine, and e-liquid prepared in our laboratory-containing 25 mg/mL nicotine) were run on gas chromatography (GC)-MS. The GC-MS system is composed of several units, including an auto-injector and auto-sampler units and a gas chromatograph (Clarus 600) connected to a single quadruple MS. A software program (TurboMass) was used for GC/MS in the post-analysis processing of our data. Elite 5 MS (30 m × 0.25 mm i.d., 0.25 μm film thickness) and GC column were used in our study for samples separation (Perkin Elmer, USA). Helium (a carrier gas), kept at a constant pressure mode (65.2 kPa) at a rate of 1.0 ml per minute, was used in the study. A gradient temperature program was carried out for sample separation. The temperature of the oven was 50^°^C for 2 minutes, increased to 150^°^C at 25^°^C/minute for 2 minutes, and then elevated to 300^°^C at 25^°^C/minute, then ramped with the grade 25^°^C/minute and held for 2 minutes. The total run time was 16 minutes. The ion source temperature was set at 240^°^C, while the injector temperature was set at 280^°^C and the interface temperature was 220^°^C. The obtained data mass spectra were found to be in the range of 40-600 m/z, and the ionization voltage was 70 eV.

The mice serum samples were prepared using the method of protein precipitation. Briefly, 50 μl of each serum sample was mixed with an amount of 25 μl of hydralazine as an internal standard (200 μg/mL) and 3.25 ml of methanol, and then the resultant was mixed using a vortex for 2 minutes. Centrifugation was carried ou at 10000 rpm for 10 minutes. Then, supernatant solution (350 μl) from each sample was taken to a sample vial, and 7 μl of each sample was injected into the UPLC/MS/MS for analysis.

In this study a validated UPLC-MS/MS (Waters Acquity, Milford, MA, USA) was carried out to determine the concentration of nicotine and cotinine in mice serum. The chromatographic procedures involved the use of a BEH HSS T3 C18 column (100x2.1 mm, 1.7 μm) with acetonitrile as a mobile phase and 0.1% formic acid (2:98 v/v) in an isocratic elution running at 0.25 ml/minute flow rate in a total run time of 3 minutes. The internal standard used in our work was hydralazine. The eluted compounds were detected using tandem MS using a TQ detector (Waters Corp., Milford, MA) supplied with an ESI source running in the mode of the positive ionization mode.

### Statistical analysis

GraphPad Prism® software was used. The Student’s t-test was used to determine whether there were any significant changes in the level of nicotine and cotinine in the serum in male and female mice, following the 4-week inhalation of e-cigarette vapors containing nicotine.

**Figure 1:**
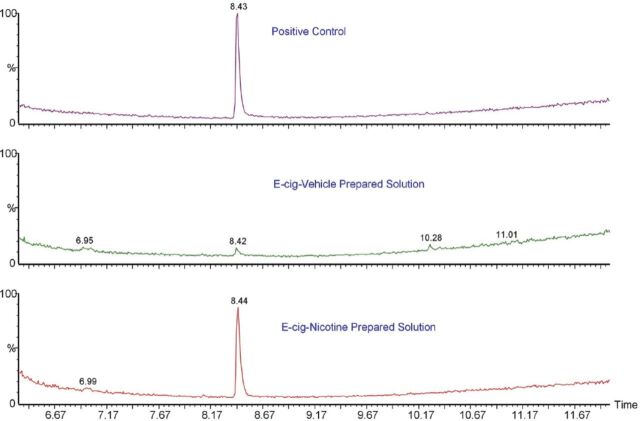
- Gas chromatography chromatograms showing the peaks of nicotine in: positive control (e-liquid-containing 25 mg/mL nicotine of a brand), e-liquid-containing 0 mg/mL nicotine, and e-liquid-containing 25 mg/mL nicotine in our prepared e-liquid.

**Results.** Gas chromatography-MS detected the nicotine area under the curve (AUC) in 3 solutions: I) positive control (e-liquid-containing 25 mg/mL nicotine of a brand); II) e-liquid-containing 0 mg/mL nicotine; and III) e-liquid-containing 25 mg/mL nicotine in our prepared e-liquid. The results showed a similar AUC of nicotine in both positive control and e-liquid-containing 25 mg/mL nicotine in our prepared e-liquid (Figure 1). This provides conformity information regarding the accuracy of our e-liquid preparations.

**Figure 2:**
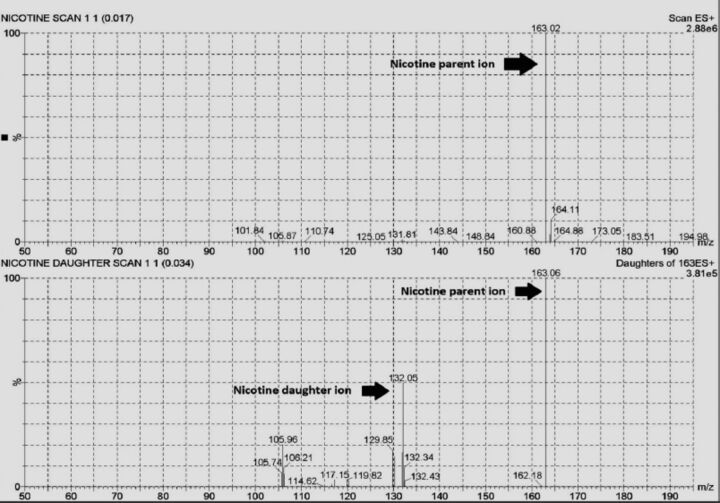
- Selection of ionization pairs (m/z) was shown as follows: nicotine= 163.06→132.05 (cone voltage: 34 V, collision energy: 20 V).

**Figure 3:**
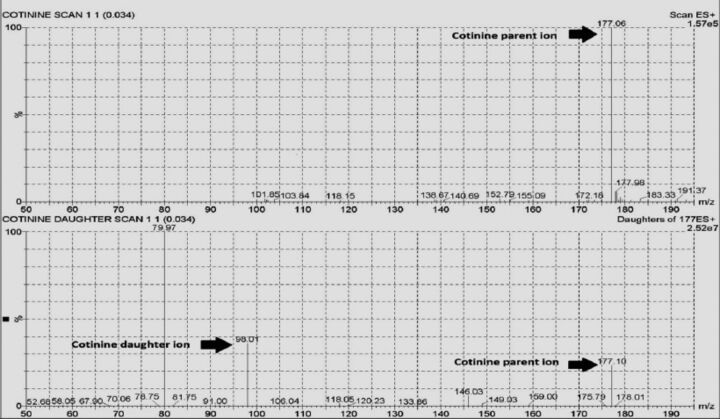
- Selection of ionization pairs (m/z) was shown as follows: cotinine= 177.1→98.01 (cone voltage: 22 V, collision energy: 18 V).

**Figure 4:**
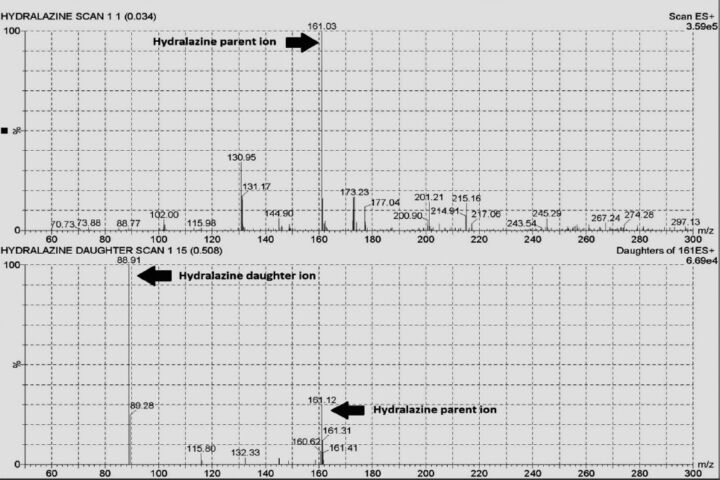
- Selection of ionization pairs (m/z) was shown as follows: hydralazine= 161.03→88.9 (cone voltage: 30 V, collision energy: 32 V).

Multiple reactions monitoring mode was applied for quantification. Selection of ionization pairs (m/z) was reported as: nicotine= 163.06→132.05 (cone voltage: 34 V, collision energy: 20 V), cotinine= 177.1→98.01 (cone voltage: 22 V, collision energy: 18 V), and hydralazine= 161.03→88.9 (cone voltage: 30 V, collision energy: 32 V). They are all shown in Figures 2-4.

**Figure 5:**
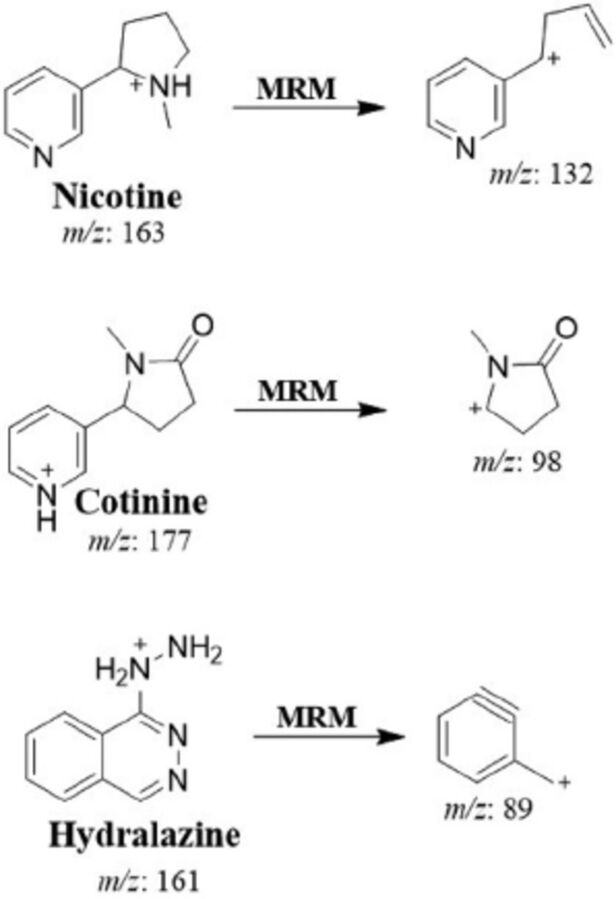
- Multiple reaction monitoring (MRM) mass transitions for nicotine (N), cotinine, and hydralazine (IS).

Multiple reactions monitoring mass transitions for nicotine, cotinine, and hydralazine are shown in Figure 5.

Several acetonitriles and 0.1% formic acid combinations were evaluated as possible mobile phases. It was determined that the combination of acetonitrile and 0.1% formic acid in an isocratic elution program (2:98 v/v) was found to be the most suitable for the separating of nicotine, cotinine, and the internal standard hydralazine. Under the described chromatographic conditions, the retention time was approximately 1.11 minutes for hydralazine, 1.77 minutes for nicotine, and 2.28 minutes for cotinine. All were eluted without any endogenous interference from the blank mice serum.

The nicotine and cotinine concentrations were measured following a published work.^7^ Extraction of the analytes was carried out using a protein precipitation method with an average recovery of 101.6±1.33 for nicotine and 100.4±0.54 for cotinine. The precision and accuracy of the developed method for nicotine and cotinine were less than 6.1. Good linearity (r^2^>0.99) was observed for cotinine and nicotine.

We further reported the LOQ and limit of detection (LOD) using signal to noise (S/N) ratio of nicotine and cotinine based on the formula: LOD = (S/N: 3.3). Instead of 3.3, LOQ as the same way is 10. Therefore, LOQ = (S/N: 10). Signal to noise ratio was 174.83 for nicotine and 89.79 for cotinine at 10 ng/mL. Thus, the LOQ for nicotine was 0.57 ng/mL and the LOD was 0.19 ng/mL, while the LOQ for cotinine was 1.11 ng/mL and the LOD was 0.38 ng/mL.

**Figure 6:**
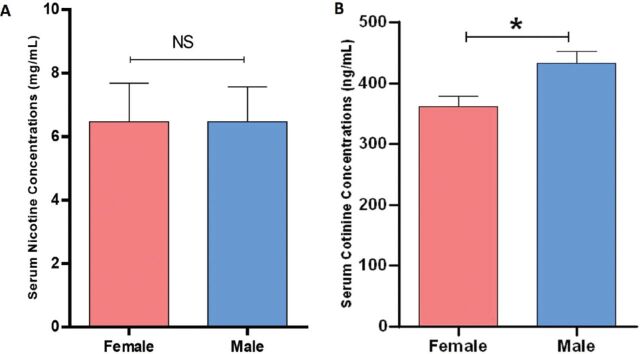
- Serum nicotine and cotinine concentrations after 4-week inhalation of e-cigarette vapors-containing nicotine in male and female mice. A) Serum nicotine concentrations following inhalation of e-cigarette vapors-containing nicotine for 4 weeks in male and female mice (NS: not significant). B) Serum cotinine concentrations following inhalation of e-cigarette vapors-containing nicotine for 4 weeks in male and female mice(^*^ *p*=0.0486).

Ultra performance liquid chromatography-MS/MS detected nicotine in male and female mice inhaled e-cigarette vapors containing 25 mg/mL nicotine for 4 weeks. The unpaired t-test showed no significant differences in the levels of nicotine between male and female mice following the 4-week inhalation of e-cigarette nicotine-containing vapors (Figure 6A). However, UPLC-MS/MS detected cotinine in male and female mice exposed to e-cigarette vapors-containing nicotine for 4 weeks. An unpaired t-test showed that the serum cotinine level in male mice was higher than female mice following the 4-week inhalation of e-cigarette vapors-containing nicotine (Figure 6B).

## Discussion

This study aimed to partially validate a sensitive UPLC-MS/MS methodology for identifying and quantifying nicotine and cotinine in mice blood samples. To achieve a highly sensitive methodology for the quantification of cotinine and nicotine, the UPLC tandem MS/MS conditions and extraction methods were optimized and used. Our study utilized an electronic delivery system to study whether a specific concentration of nicotine (25 mg/mL) and cotinine can be detected in the serum of a specific mice model (C57BL/6J mice) following 4-week inhalation of e-cigarette vapors and compare the results obtained from male mice with those from female mice to investigate if gender differences play a significant role.

Previous studies found that nicotine can be detected in plants, tobacco, cigarette smoke, vapors, e-liquids, and gases using GC.^11-14^ It has been found that nicotine in tobacco leaves was detected using GC-MS.^12^ Another prior study also detected nicotine in gases containing nicotine using GC.^11^ It was found that nicotine was detected in cigarette smoke and tobacco using GC- flame-ionization detection.^13^ This study findings were in agreement with a prior study demonstrating that nicotine was detected in the puff and e-liquids of e-cigarettes using GC-MS.^14^

Studies validated that LC-MS/MS was able to detect nicotine in humans.^15-17^ A prior study reported nicotine levels in smokers’ urine LC-MS/MS with metabolite recovery of 76-99%.^16^ Another study determined cotinine and nicotine concentrations in the serum of humans treated with nicotine transdermal delivery system using cotinine and nicotine isotope labeled as internal standards in LC-MS/MS.^15^ Byrd et al^18^ validated and developed a rapid LC-MS/MS to detect cotinine and nicotine in smokers’ serum. The recovery values for nicotine and cotinine known samples were 95-116% and 93-94%. A partial validation approach was used to detect cotinine in human saliva after an extension of this methodology. The study showed that LC-MS/MS had improved precision and accuracy for detection of nicotine as compared with radioimmunoassay methodology.^18^

Another study carried out by Kaisar et al,^10^ detected nicotine and cotinine in C57BL/6J mice plasma using UPLC-MS/MS technology following exposure to 3R4F research cigarettes 6 times/day (2 cigarettes/hour) for 14 or 7 days. The study found that a mass spectrometer with positive ion mode resulted in transitions of m/z= 177.2→98.01 for cotinine and 163.2→132.1 for nicotine. Lower LOQ in this study was determined at 3 ng/mL in brain and plasma samples of mice. This study reported that the range of nicotine concentrations van be detected was 3-200 ng/mL (r>0.995) for nicotine, while the range was 3-600 ng/mL (r>0.995) for cotinine. The transition values of cotinine and nicotine in the Kaisar et al^10^ study were approximately similar to another study by Jin et al^19^ in which U937 macrophages samples were exposed to nicotine.

Additionally, nicotine and cotinine were successfully detected in human tissues using LC/MS-MS.^17^ This indicates that LC-MS/MS is a valid technology for the detection of both cotinine and nicotine in human tissues, serum, and urine. Our study validated hydralazine as the internal standard for both nicotine and cotinine in the serum of mice exposed to e-cigarette vapors. Previous LC-MS/MS studies used isotope-labeled nicotine and cotinine as internal standards.^1,2,15^ A prior study validated acetaminophen as an internal standard of cotinine when they detected cotinine in urine using high performance LC-ESI-MS-MS technology.^20^

Our study detected the concentrations of cotinine and nicotine in the serum of both genders of C57BL/6J mice after they inhaled e-cigarette vapors containing nicotine for 4 weeks. The metabolic rate properties in each individual were varied, which may cause variations in cotinine concentrations. In a 6-month study, it was found that cotinine was at 243±14 ng/mL after inhalation of e-cigarette vapors (1 hour/day, 5 days/week, for 6 months) using female CD1 mice.^2^ In addition to the individual metabolic rate, the strain of animals may also play a role in nicotine metabolism. Another study reported cotinine at 3.95±0.70 μM in the plasma of male and female C57BL/6 mice after a 2-week inhalation of vapors-containing nicotine (3 hours/day for 14 days).^21^ Interestingly, plasma cotinine levels in regulating cigarette smokers approximately ranged between 250-300 ng/mL. However, it has been ranged up to 900 ng/mL in tobacco smokers.^22-24^

Here, we found a marked elevation in cotinine levels in male mice as compared with female mice. This finding is consistent with the previous study which showed that cotinine levels were higher in male smokers than female smokers in moderate to heavy smokers.^25^ This study suggested that this increase in cotinine levels was probably due to increased puff volume, body weight, and nicotine metabolism rate. However, another study found that cotinine levels were higher in female mice compared to male mice after subcutaneous nicotine administration suggesting that corticosterone hormone and stress play a significant role in this difference.^26^ The plasma/serum nicotine levels depend on many factors, including duration and frequency of inhalation, the number of cigarettes smoked, the concentration of nicotine, strain, age of the models, time of blood collection, and others. It was reported that nicotine levels in plasma were low after cigarette smoking at 0 minutes, but they can be raised over time.^22,27-29^ As the blood was collected ~3-5 minutes after e-cigarette exposure, the serum nicotine levels in our study are congruent with those in the previous study. While a previous study^30^ found significant changes in plasma nicotine levels between male and female rats, we did not identify significant differences between male and female mice. These findings may be because previous studies used intravenous nicotine administration whereas we used the inhalation exposure system in this study. Another reason could be attributed to differences in animal strain (Sprague-Dawley rats vs. C57BL/6J mice).

### Study limitations

Low sample size was used in our study. Future work are warranted to further confirm our findings using larger sample size.

In conclusion, the presented method is simple and has acceptable sensitivity. Thus, it is recommended to be applied in forensic and clinical toxicology laboratories. Our data provide evidence that the electronic delivery system was successful in mimicking the human physiological exposure to e-cigarette vapors-containing nicotine. Our study indicated that gender plays a significant role in nicotine metabolism in nicotine dependence models. Future studies are required to investigate cotinine concentrations in males and females, nicotine dependence models as well as determine the factors that affect cotinine concentrations.
